# Spiritual Needs of Lung Cancer Patients and Their Relation to Psychological Distress and Quality of Life

**DOI:** 10.7759/cureus.20225

**Published:** 2021-12-07

**Authors:** Evangelos C Fradelos, Eleni Albani, Ioanna V Papathanasiou, Paraskevi-Maria Prapa, Effrosyni Tsomaka, Vissarion Bakalis, Sofia Artemi, Maria Lavdaniti

**Affiliations:** 1 Department of Nursing, University of Thessaly, Larissa, GRC; 2 Department of Nursing, University of Patras, Patras, GRC; 3 Community Nursing Lab, Department of Nursing, University of Thessaly, Larissa, GRC; 4 Internal Medicine, Athens Hospital for Chest Diseases “Sotiria”, Athens, GRC; 5 Psychiatry, Athens Hospital for Chest Diseases “Sotiria”, Athens, GRC; 6 Intensive Care Unit, General Hospital “Elpis”, Athens, GRC; 7 Nurisng, International Hellenic University, Thessaloniki, GRC

**Keywords:** health-related quality of life, psychological distress, lung cancer, spiritual needs, spirituality

## Abstract

Background

This study aimed to investigate the spiritual needs of patients suffering from lung cancer in relation to their mental health and quality of life.

Methodology

A cross-sectional quantitative study design was employed to investigate 110 lung cancer patients receiving chemotherapy. A four-part self-assessment instrument was used to gather the data comprising a sheet containing demographic and clinical information, Spiritual Needs Questionnaire, The Depression, Anxiety, and Stress Scale-21 Items, and the 12-item Health Survey. Descriptive inferential statistics were applied.

Results

Of the 110 patients, 71.8% were men, the mean age was 64.25 (±9.3) years, and 71.8% were married. In total, 40.9% of the patients were retired, and 92.7% had a public insurance company. Regarding education, 30% were primary school graduates and 31.8% were high school graduates. Regarding the clinical features of the sample, 23.6% of the patients had small-cell carcinoma, 71.9% had non-small-cell carcinoma, and 4.5% had large-cell carcinoma. Spiritual needs have a negative effect on the mental health component of quality of life (p < 0.001) and can increase psychological distress in lung cancer patients.

Conclusions

In contrast to the findings of other international studies, spiritual needs appeared to be lower; however, similar to other studies, spiritual needs increased in those suffering from depression and anxiety. Moreover, the subtype of lung cancer also appeared to play a role.

## Introduction

Lung cancer is one of the most prevalent types of cancer and one of the major causes of death worldwide [1]. In Greece, it is the most common cause of cancer-related deaths in both sexes. The diagnosis of lung cancer leads to significant changes in a person’s life due to the short survival time. Psychological symptoms such as depression, loss of interest, and suicidality are often present both during the diagnosis and the course of the disease [2]. Studies have reported a high incidence of depression (33-44%) among patients suffering from lung cancer [2].

Quality of life is defined by the World Health Organization as “an individual’s perception of their position in life in the context of the culture and value systems in which they live in relation to their goals, expectations, standards, and concerns.” Multiple factors affect the quality of life of patients suffering from lung cancer [2]; moreover, psychological distress is associated with reduced functionality and reduced quality of life in end-stage patients [3].

The most common symptoms experienced by patients with end-stage lung cancer include fatigue and anorexia. Recent studies have attempted to investigate the symptoms experienced by patients with end-stage cancer. Other topics of interest include the impact of lung cancer on the quality of life of patients, the effect of the symptoms occurring from the treatment on the quality of life, and depression [4,5].

In recent years, the scientific community, especially health scientists, has been increasingly concerned with spirituality and its contribution to the treatment of various illnesses [6]. Undoubtedly, for many people, spirituality and religiosity constitute a significant approach to managing stress and the difficulties of everyday life. Moreover, researchers have attributed to spirituality the ability to help individuals illustrate difficulties, illnesses, and even death. It is experienced in the last moments of one’s life, that is, when people try to escape uncertainty and gain a sense of fullness. It is because of this constant search for healing and fulfillment that Puchalski (2004) called spirituality a human aspect [7,8].

The new models of healthcare services that consider all aspects of the patient’s individuality and the mental, spiritual, and social needs appear to contribute significantly to the patient’s adaptation to chronic diseases and better recovery. The recognition and satisfaction of spiritual needs are crucial and imperative for the multidimensional care of cancer patients. The effect of mental distress on not only the mental and social levels but also the adaptation to and outcome of the disease implies the importance of conducting further research with reliable tools to assess patients’ mental needs and general conditions [8,9].

In particular, patients with lung cancer experience significantly greater psychological distress compared to patients with other cancers, and respiratory symptoms are a major source of stress in this patient population. It has been stated that spirituality is related to the psychological adjustment to illness, and several studies have indicated a link between participation in religious activities and lower mortality [10].

Moreover, people diagnosed with cancer apprehend the importance and contribution of their spirituality in the planning of their treatment, experience of survival, and preparation for their probable death. The majority of these patients expect that oncologists and other health professionals examine their spirituality and incorporate their spiritual strengths and needs into their treatment plan. The need to meet the mental, social, and spiritual requirements of patients with cancer, along with their physical requirements, throughout their care is well documented. This stress on spirituality results not only from the desire of the patients themselves but also from the benefits to the quality of their life. Therapists’ inquiry of the spirituality and spiritual needs of such patients is also crucial to establishing trust with the patient and ensuring that the treatment is planned according to the patients’ beliefs and values. Failure to address spirituality can lead to poorer treatment outcomes, increased non-compliance with the treatment plan, and a lack of help for patients in finding effective coping mechanisms. The dissatisfaction with spiritual needs has been associated with lower patient satisfaction [11], poorer quality of life, and higher end-of-life costs [12].

Despite the key role of spiritual needs in the holistic evaluation of patients, in practice, health professionals may avoid discussing them due to lack of time, inadequate training, poor comprehension of spirituality, and even feelings of personal vulnerability in an area where definitive solutions are rare. Moreover, because modern spirituality is eclectic, individualistic, and in a sacred-cosmic continuum, defining spiritual needs and spiritual care is difficult. Thus, for the common understanding of the term “spiritual care,” defining it is necessary. Murray et al. (2004) defined spiritual needs as “the needs and expectations that individuals have for finding meaning, purpose, and value in their lives. Such needs may be specifically religious, but even people without religious faith or who are not members of organized religion have belief systems that give meaning and purpose to their lives” [13]. Thus, spiritual care means “caring for the identification and response to the spiritual needs of individuals when faced with trauma, illness, grief, and suffering.” This includes addressing the need for meaning, self-esteem, and self-expression, as well as offering the support of faith, prayer, sacraments, and religious practices [14].

In Greece, many studies have been conducted on the spirituality and religiosity of cancer patients and the impact these may have on various health-related parameters [15,16]. However, research on the spiritual needs of inpatients is limited, and the results of such studies are contradictory. Further, Greek and international literature exploring the relationship between quality of life, mental stress, and spiritual needs among patients with lung cancer is limited. Hence, this study aimed to investigate the spiritual needs of patients suffering from lung cancer, as well as the relationship of those spiritual needs to their mental health and quality of life. Specifically, we aimed to assess the following research questions: (1) Do lung cancer patients experience spiritual needs, and to which extend?; (2). Is there a relationship between spiritual needs and the quality of life of those patients?; and (3) Is there a relationship between spiritual needs and psychological distress?

## Materials and methods

This was a cross-sectional quantitative study. A self-administered instrument was used to gather the data.

Study sample

The study involved 110 lung cancer patients who were treated using chemotherapy in a day clinic of a general hospital in Athens. The inclusion criteria were as follows: (a) age 18 years or older; (b) Greek speakers; (c) lung cancer diagnosis of any subtype; (d) treatment with chemotherapy or other methods; (e) satisfactory level of cooperation; and (f) ability of capacity. Data were collected from April 2020 to May 2020.

Study instruments

Spiritual Needs Questionnaire

The Spiritual Needs Questionnaire (SpNQ) consists of 27 items and four main factors, namely, religious needs, existential needs, needs for inner peace, and giving/generativity needs. To measure the patients’ spiritual needs, we used the 19-item version of the SpNQ. It differentiates among the following four main factors: (1) Religious needs: praying for and with others, praying alone, participating in a religious ceremony, reading spiritual/religious books, and turning to a higher presence (i.e., God, angels); (2) existential needs (reflection/meaning): reflecting on one’s life, talking with someone about the meaning of life/suffering, dissolving open aspects in life, talking about the possibility of life after death, etc.; (3) need for inner peace: wishing to dwell in places of quietness and peace, plunging into the beauty of nature, finding inner peace, talking with others about fears and worries, and turning to someone with a loving attitude; (4) need for active giving/generativity: harboring the autonomous intention to give and actively giving solace to someone, passing along one’s own life experiences to others, and being assured that life was meaningful and of value. All the items were scored concerning self-ascribed importance on a four-point scale, ranging from disagreement to agreement (0: not at all; 1: somewhat; 2: very; 3: extremely) [17,18].

The Depression, Anxiety, and Stress Scale-21 Items

The Depression, Anxiety, and Stress Scale-21 Items is a 21-question assessment that was developed to measure the degree of depression, anxiety, and stress (three separate but interrelated areas) experienced by an individual. It includes seven questions for each subscale, and the scores are calculated by adding up the scores for every item in the subscales. For depression, the scale measures the levels of dysphoria, hopelessness, devaluation of life, self-deprecation, lack of interest/involvement, anhedonia, and inertia. For anxiety, the scale measures autonomic arousal, skeletal muscle effects, situational anxiety, and subjective experience of anxious affect. Lastly, for stress, the scale measures difficulty in relaxing, nervous arousal, being easily upset/agitated, being irritable/over-reactive, and impatience. Respondents are asked to rate each item on a four-point scale based on what they had experienced during the past week [19].

12-Item Health Survey

The 12-item Health Survey (SF-12) was developed as a shorter alternative to the 36-Item Short-Form Health Survey for use in large-scale studies, particularly when overall physical and mental health, rather than the typical eight-scale profile, are the outcomes of interest. All 12 items are used to calculate the physical and mental component summary scores (PCS-12 and MCS-12). This survey has been translated into Greek; its reliability and validity were established using a sample of 1,007 adults living in the greater Athens area [20].

Statistical analyses

Descriptive statistics as well as analyses of variance, first-order correlations, and regression were computed with SPSS version 25.0 (IBM Corp., Armonk, NY, USA). We considered p < 0.05 as significant; for correlation analyses, we chose a significance level of p < 0.001. Concerning the strength of the observed correlations, we regarded r > 0.5 as a strong correlation, r between 0.3 and 0.5 as a moderate correlation, r between 0.2 and 0.3 as a weak correlation, and r < 0.2 as a negligible correlation.

Quantitative variables were presented as means (±SD) and qualitative variables as absolute and relative frequencies. We performed bivariate analyses between sociodemographic characteristics and the scores on SpNQ. Student’s t-test and analysis of variance (ANOVA) were used to determine the association between categorical and continuous variables, and Pearson’s correlation coefficient was used to determine the correlation between continuous variables. All statistical analyses were conducted using SPSS, and p-values of 0.05 were considered statistically significant.

Compliance with ethical standards

The study received ethical approval from the ethics committee of Athens General Hospital for Chest Diseases “Sotiria” where the study was conducted (8676.78.01.04.2020). Written informed consent was obtained from all participants. We assert that all procedures contributing to this work complied with the ethical standards of the relevant national and institutional committees on human experimentation and with the Helsinki Declaration of 1975, as revised in 2008.

## Results

Of the 110 patients, 71.8% were men, the mean age was 64.25 (±9.3) years, and 71.8% were married. In total, 40.9% of the patients were retired, and 92.7% had a public insurance company. Regarding education, 30% were primary school graduates and 31.8% were high school graduates. Regarding the clinical features of the sample, 23.6% of the patients had small-cell carcinoma, 71.9% had non-small-cell carcinoma, and 4.5% had large-cell carcinoma. The median duration of illness was 14 months, and 67.3% of the patients had no other health problems. The mean hematocrit level was 37.7% (±5.1%), the mean hemoglobin level was 13.1 g/dL (±4.2 g/dL), and the mean white blood cell count was 8,307/µL (±5,201/µL) (Table 1).

**Table 1:** Clinical and demographic characteristics of patients. SD: standard deviation; Ht: hematocrit; Hb: hemoglobin; WBC: white blood cell

		N (%)
Gender	Male	79 (71.8%)
	Female	31 (28.2%)
Age (mean ± SD)		64.25 ± 9.3
Marital status	Married/cohabitation	79 (71.8%)
	Unmarried	31 (28.2%)
Living arrangement	Alone	25 (22.7%)
	Cohabitation	85 (77.3%)
Educational status	Mandatory/secondary	97 (88.2%)
	Higher education	13 (11.8%)
Working	Yes	28 (25.5%)
	No	82 (74.5%)
Area of residence	Urban	94 (85.5%)
	Semi-urban	12 (10.9%)
	Rural	4 (3.6%)
Type of cancer	Small cell	26 (23.6%)
	Non-small cell	79 (71.8%)
	Large cell	5 (4.5%)
Duration in months (mean ± SD)		14.05 ± 16.76
Metastasis	No	51 (46.4%)
	Yes	59 (53.6%)
Type of therapy	Chemotherapy	60 (54.5%)
	Combination	50 (45.5%)
Comorbidities	No	74 (67.3%)
	Yes	36 (32.7%)
Laboratory values (mean ± SD)	Ht (%)	37.7 ± 5.1
	Hb (g/dL)	13.1 ± 4.2
	WBC (/µL)	8,307 ± 9,525
Quality of life (mean ± SD)	Physical health	38.1 ± 9.9
	Mental health	45.6 ± 11.8
Psychological distress (mean ± SD)	Depression	4.55 ± 5
	Anxiety	3.84 ± 4.1
	Stress	5.21 ± 5.01

Spiritual needs of lung cancer patients

To answer the first research question regarding the experience of spiritual needs in lung cancer patients we calculated mean scores of the SpNQ. In addition, we examined the relationship between spiritual needs and patients’ clinical, social, and demographic characteristics.

The analysis of the SpNQ tool showed that the features depicted relatively low values, considering the theoretical range of the tool. The statistical analysis of the features is presented in Table 2, with the mean values (mean response for each feature) ranging from 0.690 (a feature of existential needs) to 1.036 (a feature of need for giftedness and creativity). There was a relatively good variance in relation to the mean values (SD: 0.59-0.78). Regarding the relationship of spiritual needs with the clinical, social, and demographic characteristics of the sample, the following was observed: Age was found to be negatively related to religious needs (r = 0.196, p < 0.05) and inner peace needs (r = 0.192, p < 0.05). Moreover, patients suffering from small-cell cancer experienced more existential needs (F (2.107) = 3.315, p = 0.040) compared to those with non-small-cell carcinoma least significant difference (LSD) (p = 0.018). Parallel differences were discovered between these groups with regard to the giving/generativity needs (F (2.107) = 4.253, p = 0.017) LSD (p = 0.004), and spiritual needs total score (F (2.107) = 3.152, p = 0.047) LSD (p = 0.019).

**Table 2:** Spiritual needs of lung cancer patients. SpNQ: Spiritual Needs Questionnaire; SD: standard deviation

	Mean ± SD
Religious needs	0.77 ± 0.75
Existential needs	0.69 ± 0.64
Inner peace needs	0.89 ± 0.68
Giving/generativity needs	1.03 ± 0.78
SpNQ-27 total	0.92 ± 0.59

To answer the second and the third research question, first, we performed a correlation and regression analysis of spiritual needs, quality of life, and psychological distress of lung cancer patients.

In Table 3, the correlations of the dimensions of SpNQ-27 with the physical and mental health factors of the SF-12 quality of life measurement tool are depicted. Almost all dimensions showed a statistically significant negative correlation with the mental health dimension of quality of life, except for the dimension of inner peace needs. No correlation was found between physical health and spiritual needs. During the examination of the relationship between spiritual needs and mental stress, the following was observed: The dimension of existential needs was positively correlated with the scores for depression (r = 0.218, p = 0.006) and stress (r = 0.285, p = 0.001). The same was observed for the overall score of spiritual needs, which was found to be positively related to the scores for depression (r = 0.210, p = 0.017) and stress (r = 0.279, p = 0.002). Finally, the dimension of inner peace was correlated with stress (r = 0.200, p = 0.028). The findings of multiple regression analysis indicated that spiritual needs have a negative effect on the mental health component of quality of life (b = -8.65 (9.69), p = 0.001) and can increase psychological distress in lung cancer patients. The detailed results of the multiple regression analysis are presented in Table 4.

**Table 3:** Correlation of spiritual needs, quality of life, and psychological distress. SF-12: 12-item Health Survey; HrQoL: health-related quality of life; DASS-21: The Depression, Anxiety, and Stress Scale-21 Items; SpNQ: Spiritual Needs Questionnaire

	SF-12 HrQoL		DASS-21		
	Physical health	Mental health	Depression	Anxiety	Stress
Religious needs	r = -0.002, p = 0.704	r = -0.219, p = 0.033	r = 0.112, p = 0.202	r = 0.077, p = 0.302	r = 0.118, p = 0.238
Existential needs	r = -0.063, p = 0.333	r = -0.293, p = 0.004	r = 0.218, p = 0.006	r = 0.183, p = 0.011	r = 0.285, p = 0.001
Inner peace needs	r = -0.123, p = 0.043	r = -0.179, p = 0.123	r = 0.116, p = 0.169	r = 0.069, p = 0.369	r = 0.200, p = 0.028
Giving/Generativity needs	r = -0.108, p = 0.251	r = -0.194, p = 0.040	r = 0.093, p = 0.529	r = 0.046, p = 0.157	r = 0.166, p = 0.161
SpNQ-27 total	r = -0.113, p = 0.187	r = -0.306, p = 0.001	r = 0.210, p = 0.017	r = 0.147, p = 0.069	r = 0.279, p = 0.002

**Table 4:** Multiple linear regression analysis with HrQoL and psychological distress as dependent variables and spiritual needs as the independent variable, adjusted for demographic and clinical characteristics. SF-12: 12-item Health Survey; HrQoL: health-related quality of life; DASS-21: The Depression, Anxiety, and Stress Scale-21 Items; SpNQ: Spiritual Needs Questionnaire

	SF-12 HrQoL				DASS-21											
	Mental health				Depression				Anxiety				Stress			
Spiritual needs	Β(SE)	p	Lower	Upper	Β(SE)	p	Lower	Upper	Β(SE)	p	Lower	Upper	Β(SE)	p	Lower	Upper
Religious needs	7.73(3.5)	0.004	0.754	14.717	-4.80(1.48)	0.001	-7.764	-1.855	-3.18(1.26)	0.001	-5.701	-0.675	-6.04(1.40)	0.001	-8.833	-3.260
Existential needs	0.5(4.20)	0.620	-7.811	8.853	-0.02(1.77)	0.724	-3.551	3.500	1.09(1.51)	0.284	-1.909	4.089	-0.13(1.67)	0.867	-3.457	3.194
Inner peace needs	5.59(2.81)	0.011	0.001	11.186	-2.64(1.19)	0.010	-5.016	-0.283	-2.00(1.01)	0.016	-4.017	0.009	-2.43(1.12)	0.008	-4.662	-0.197
Giving/generativity needs	7.58(3.16)	0.002	1.294	13.869	-4.19(1.34)	0.001	-6.852	-1.530	-2.73(1.14)	0.001	-4.999	-0.472	-4.58(1.26)	0.001	-7.091	-2.072
SpNQ-27 total	-8.65(9.69)	0.001	-47.895	-9.417	14.37(4.10)	0.001	6.234	22.517	8.53(3.49)	0.003	1.607	15.457	16.59(3.87)	0.001	8.915	24.274
	F = 2.71; p = 0.01; R^2^ = 11.7%				F = 2.99; p = 0.05; R^2^ = 12.7%				F = 2.15; p = 0.03; R^2^ = 7.2%				F = 4.66; p < 0.01; R^2^ = 21.2%			

## Discussion

The goal of this study was to evaluate the spiritual needs of lung cancer patients and their relation to the quality of life and mental symptoms of these patients. One of the main findings of the study was that the patients reported few unmet spiritual needs, which can be explained by the close religious ties that exist in Greek society. Another significant finding was the absence of statistically significant correlations between the variables under investigation.

Spirituality is a fundamental dimension of human life and an integral and universal dimension of human existence. It relies on various religious traditions, spiritual movements, value systems, and cultures. Each individual experiences and feels spirituality uniquely; it is an individual experience, independent of common beliefs or traditions. Spirituality plays an important part in health, especially in times of crisis or serious illness as it is fundamental and connects the bio-psycho-social dimensions to wholeness [21]. Spirituality is an important resource for patients experiencing chronic or life-threatening illness and loss. Health professionals should carefully manage and meet all of their patient’s physical, psychological, and spiritual needs as it is an integral part of care. They can evolve into true partners by honestly listening to their patients’ hopes, fears, and beliefs and incorporating those beliefs into their treatment plan [22].

The features were analyzed to determine the spiritual needs of lung cancer patients. Examining the mean values of the answers given for each feature of the questionnaire, we can conclude that patients show low-intensity spiritual needs. These results are similar to those of a survey in Germany involving 100 elderly people living in nursing homes [23]. In Greece, patients express their faith in God; this can be understood from their participation in various religious events. In addition, many individuals’ vivid interactions with their religious communities result in their religious and spiritual pursuits being satisfied, which may explain the aforementioned decrease [24,25].

It has been stated that the psychological symptoms of lung cancer patients in palliative care depend on the meaning the patients attach to the diagnosis of lung cancer. A new diagnosis motivates them to redefine their values and priorities in their lives. With regard to their religious beliefs, a study found that people who evaluated the diagnosis positively (as a trial given from God) had reduced stress and depression, while those who faced it negatively (as a punishment) had the opposite result [26]. In other studies, a comparison between the spiritual concerns of those diagnosed and those not diagnosed with lung cancer showed that the former experience more spiritual concerns [27]. Special cultural and religious habits should be considered with regard to the spiritual needs of people diagnosed with lung cancer. In a study on the Muslim population, spiritual needs were found to increase compared to other studies; this may be related to their prayer ritual. Furthermore, in other Islamic cultures, religious needs tend to be more significant than other spiritual needs [28]. Surveys have shown that spiritual needs appear to concentrate more on religious beliefs even though these depend on the religious structure of each community. Moreover, death and the discussion around it have dominated recent literature, resulting in the finding that spirituality and the feeling of hope help people with religious beliefs manage this issue [29].

In the literature, a connection between spirituality and the quality of life has been reported. Besides, spirituality tends to be part of the common belief about well-being; people suffering from cancer seem to connect spirituality and religious coping with a better quality of life. Several studies have also indicated that spirituality decreases anxiety, depression, and negative feelings and that the quality of life appears to improve due to psychological interventions. According to our results, unmet spiritual needs can lead to poor quality of life in the mental health component and increased depression, anxiety, and stress. These results are in agreement with various international studies that have indicated the negative effects of unmet spiritual needs on a patient’s health, highlighting the necessity of spiritual care in cancer trajectory [30].

Among the strengths of this study is the novelty of the study, especially in the Greek context. Despite its unique methodology and findings, this study has certain limitations. First, the sample selected for this study was small and from a single hospital located in Athens. More studies in other facilities examining the topic should be conducted. Hence, our findings cannot be generalized without considering a sample that includes patients from around the country. Second, a larger sample size would have made the study more representative. Finally, a cross-sectional approach was employed, and no researcher can confidently claim causal relations based on cross-sectional studies. The results of the present study should be confirmed through a longitudinal investigation. Finally, this study was conducted during the first pandemic wave in Greece, which could have an impact on our results, especially regarding the psychological distress of the patients as cancer patients are considered to be a vulnerable poppulation concerning coronavirus disease 2019.

## Conclusions

Spirituality is an important aspect for many people, especially for those facing a serious and potentially life-threatening disease such as lung cancer. Greek lung cancer patients experience few spiritual needs compared to other studies, yet the inner peace and giving/generativity needs were increased in our study population. Inner peace needs were associated with poor quality of life (mental health domain) as well as increased psychological distress. The results of this study can help healthcare professionals to understand when, for whom, and why spiritual needs must be assessed and the benefits spiritual care integration may have on patients’ mental health and quality of life. 
